# Developmental Mechanisms in Articular Cartilage Degradation in Osteoarthritis

**DOI:** 10.1155/2011/683970

**Published:** 2010-12-29

**Authors:** Elena V. Tchetina

**Affiliations:** Institute of Rheumatology, Russian Academy of Medical Sciences, Kashirskoye Shosse 34A, Moscow 115522, Russia

## Abstract

Osteoarthritis is the most common arthritic condition, which involves progressive degeneration of articular cartilage. The most recent accomplishments have significantly advanced our understanding on the mechanisms of the disease development and progression. The most intriguing is the growing evidence indicating that extracellular matrix destruction in osteoarthritic articular cartilage resembles that in the hypertrophic zone of fetal growth plate during endochondral ossification. This suggests common regulatory mechanisms of matrix degradation in OA and in the development and can provide new approaches for the treatment of the disease by targeting reparation of chondrocyte phenotype.

## 1. Introduction

 Osteoarthritis (OA) is the most common joint disease, which is associated with a risk of mobility disability. It affects approximately 12% of the aging Western population, while a quarter of people aged over 55 have an episode of persistent knee pain [1]. The pathology of OA involves the whole joint and is associated with focal and progressive hyaline articular cartilage loss, concomitant sclerotic changes in the subchondral bone, and the development of osteophytes. Soft tissue structures in and around the joint including synovium, ligaments, and muscles are also involved [2].

 OA affects predominantly articular cartilage, which degrades by gradual loss of its extracellular matrix (ECM) composed mainly of aggrecan and type II collagen. Loss of large proteoglycan aggrecan decreases cartilage compressive stiffness and precedes the damage to collagen fibrillar network, which is responsible for tensile properties of the tissue [3]. Aggrecan degradation is associated with upregulation of aggrecanases a disintegrin and metalloprotease with thrombospondin motifs (ADAMTS-) 4 and 5 as well as matrix metalloproteinases (MMPs) [4]. The excessive cleavage of type II collagen in OA is assumed to be caused by the upregulation of the synthesis and activities of collagenases [5–7], in particular MMP-13 [8–10]. Presently, it is believed that articular cartilage destruction in OA results from excessive loading, age-related changes, and metabolic imbalance in the tissue [11–13]. 

 OA also exhibits features of a systemic disease as it has been shown to involve vascular pathology [14, 15] as well as T-cell immune response [16, 17] associated with upregulation of cytokines such as interleukin (IL-) *β* and tumor necrosis factor (TNF)*α* [3, 18], which aggravate cartilage resorption [19]. As the mechanism of OA development is not completely understood, the disease manifestations, which are associated with cartilage resorption and inflammation, suggest a treatment involving inhibition of proinflammatory cytokines or MMP activity to prevent matrix destruction. However, it does not result in disease modification and produces severe side effects [20, 21].

 Articular cartilage degeneration in OA is also associated with changes in chondrocyte phenotype [13, 22, 23]. Specifically, these changes resemble those observed during chondrocyte differentiation in endochondral ossification and are characterized by cell cloning, expression of differentiation-related genes such as parathyroid hormone-related peptide (PTHrP) [24], type X collagen [25–27], annexins and alkaline phosphatase (ALP) [28, 29], osteocalcin [30], matrix calcification [31, 32], as well as apoptotic cell death of terminally differentiated chondrocytes [33, 34]. All these cellular changes including increased cleavage of type II collagen by MMP-13 are also associated with chondrocyte hypertrophy observed in the growth plate [35]. This suggests that, as articular cartilage shares a common embryological origin with the epiphyseal growth plate [36], destruction of cartilage matrix in OA may involve some of the same cellular and regulatory mechanisms that govern normal chondrocyte terminal differentiation and ECM resorption in skeletal growth and repair [22]. 

 The aim of this paper is to summarize current evidence supporting the involvement of molecular mechanisms observed in the course of chondrocyte progression through the growth plate in cartilage matrix destruction in OA.

## 2. Zonal Gene Expression in Epiphyseal Growth Plate

 A central process in endochondral bone formation is a progressive differentiation of proliferating matrix assembling chondrocytes to growth-arrested hypertrophic cells. This involves remodeling and mineralization of the cartilage matrix and leads eventually to its subsequent replacement by bone. 

 Primary mammalian growth plate physis is structurally organized and can be divided into zones, namely, the resting, proliferative, and hypertrophic. Resting zone chondrocytes show very limited cell division evidenced by low proliferating cell nuclear antigen (PCNA) expression [37]. They elaborate an extensive extracellular matrix, which is composed predominantly of type II collagen and proteoglycan aggrecan; however, it also contains other collagen types VI, IX, XI, link protein, and small leucine-rich proteoglycans (SLRPs) such as decorin and fibromodulin [37]. Expression of several regulatory growth factors, such as bone morphogenetic proteins (BMPs-) 3, 5, 7, fibroblast growth factor (FGF-) 2, and transforming growth factor (TGF)*β*1–3 has been detected in this zone as well [37–43]. 

 In contrast to resting zone, proliferative zone chondrocytes actively divide, which is evidenced by the expression of cyclins [35, 44] and the presence of PCNA positive cells [45]. They produce long columns of flattened cells and express hyaline ECM similar to resting zone chondrocytes. The space for the cells newly formed in the course of cell division is generated by the matrix-degrading activity of collagenases MMP-13, MT1-MMP [46, 47], and other MMPs such as MMP-3 [48]. These cells also express proliferation-specific growth factors, namely, TGF*β*1–3, FGF-2 [35, 43, 49, 50], PTHrP, insulin growth factor (IGF-) I and II [35, 51–53], a cell death inhibitor that regulates apoptosis Bcl-2 (B-cell lymphoma-2) [54], and transcription factor Sox9 (SRY-type high-mobility-group box transcription factor 9) [35, 55]. Although PTHrP [56], TGF*β*2, and FGF-2 [57, 58] have been reported to stimulate MMP-13 expression in rodents, in the early proliferative zone of the growth plate, their expression does not induce significant matrix loss probably due to the lack of gelatinase (MMP-2 and -9) expression [35, 59]. 

 Cessation of cell division in the growth plate is associated with upregulation of cell cycle inhibitors p18, p19, and p21 [60], growth arrest and DNA damage-inducible (GADD) 45beta gene [61, 62], as well as apoptosis inhibitors Bcl2 and Bag1 (Bcl2-associated athanogene 1), a Bcl2-binding protein capable of enhancing Bcl2 activity [42, 63], and a marker of apoptosis caspase 3 [42]. At this point, chondrocytes partially resorb their extracellular matrix, enlarge, round up, and finally mature into hypertrophic cells, which express type X collagen (COL10A1), a marker of chondrocyte hypertrophy. Alkaline phosphatase shows the most pronounced expression also in hypertrophic chondrocytes [64, 65]. This phenotypic modification in growth plate chondrocytes is associated with dramatic alteration in regulatory gene expression, namely, upregulation of growth factors such as TGF*β*1 and -3 [35, 50], BMP-2, -4, -6, and -7 [39, 40, 66–68], connective tissue growth factor (CTGF) [69], vascular endothelial growth factor (VEGF) [59, 70], and Indian hedgehog (Ihh) [35, 71, 72]. Inflammation-related cytokine IL-1 expression also has been observed only in the hypertrophic chondrocytes [73].

 These regulatory growth factors are expressed in association with runt-related transcription factor (RUNX)2, which is essential both for osteoblast differentiation [74] and chondrocyte maturation during endochondral ossification [75–78], and is capable of inducing MMP-13 expression [79, 80]. 

 Expression of these growth and transcription factors is also associated with upregulation of matrix proteins, such as collagen type II (COL2A1) concomitantly with their degrading enzymes MMP-13 and gelatinases MMP-2 and -9 [35, 67]. At this time, overt type II collagen degradation occurs [46] indicating that genes for both matrix synthesis and degradation are coregulated. However aggrecan remains retained in the tissue at that time [3]. 

 In the lower hypertrophic zone, mineralization (or calcification) of residual matrix remaining after its resorption is initiated focally [3]. This involves deposition of hydroxyapatite mineral [81]. Mineralization process in the lower hypertrophic zone of the growth plate is associated with expression of osteocalcin, which is a marker of mature osteoblasts and is involved in chondrocyte mineralization and Ca^+2^ homeostasis [28]. Upregulation of ankylosis protein (Ank), which is responsible for transport of intracellular inorganic pyrophosphate to the extracellular milieu, has been also observed in this zone [82]. Mineralization is likely regulated by annexins II, V, and VI, which are highly expressed in the hypertrophic and terminally differentiated mineralizing growth plate chondrocytes and form calcium channels enabling formation of first mineral phase [83, 84]. For example, annexin V has been shown to be capable of upregulating annexins II, VI, osteocalcin, Runx2, and ALP as well as stimulating apoptotic activity in the lowest part of the growth plate [83, 85]. In contrast, TGF*β*2, which is also expressed by lower hypertrophic chondrocytes [35], is most probably involved in osteoblast formation [86].

 Therefore, chondrocyte maturation in the growth plate is associated with expression of stage-specific set of regulatory growth and transcription factors producing changes in cellular phenotype and synthesis of stage-specific extracellular matrix, which eventually degrades in the hypertrophic zone. All these cellular activities require careful and specific coordination.

## 3. Regulation of Growth Plate Chondrocyte Differentiation

 Chondrocyte differentiation is initiated in the center of the cartilaginous bone rudiment and is thought to be induced by hypoxia and/or nutrient deficiency [87]. The pace of chondrocyte differentiation is regulated by various agents including paracrine and autocrine growth factors and hormones [3, 88]. They are responsible for specific regulatory molecule expression by chondrocytes in the course of their progression through the growth plate. 

 Growth factors secreted by fetal chondrocytes are in charge of mutually exclusive processes of chondrocyte proliferation and terminal differentiation. Thus, proliferation-related growth factors such as basic fibroblast growth factor and parathyroid hormone-related peptide stimulate resting chondrocytes to proliferate and suppress terminal differentiation of hypertrophic chondrocytes [89–96]. In addition, PTHrP, in combination with Indian hedgehog, regulates chondrocyte differentiation through the establishment of a negative feedback mechanism, whereby Ihh and PTHrP can together suppress hypertrophy [97–100]. Alternatively, interactions of Ihh with syndecan 3, which serves as a growth factor coreceptor, are important for restricting mitotic activity to the proliferative zone of mammalian growth plate [101].

 Transforming growth factor betas are multifunctional molecules regulating cellular proliferation, differentiation, and extracellular matrix function [75, 102]. TGF*β* transported from apoptotic chondrocytes to the region of cell division would be expected to stimulate matrix production, delay hypertrophic differentiation, and thus maintain growth plate width [103, 104]. TGF*β*1 [105–107] and TGF*β*2 [108] each are able to suppress chondrocyte hypertrophy by coordinate inhibition of collagenase expression. This is partially associated with upregulation of PTHrP gene expression that exerts both PTHrP-dependent and PTHrP-independent effects on endochondral bone formation [105, 109, 110]. TGF*β*2 in synergy with FGF-2 has been also shown to suppress chondrocyte maturation and hypertrophy [111, 112]. 

 BMP signaling is also essential for chondrocyte progression through the growth plate [66]. Zone specific expression of various BMPs suggests their involvement in chondrocyte phenotypic changes in the course of both proliferation and hypertrophy. Thus, BMP-2 and -6 have been shown to promote chondrocyte hypertrophy by upregulation of Ihh and type X collagen expression and downregulation of FGF signaling involving Runx2 [113–118]. At the same time, BMP-2 and -9 augmented mitogenic effect of IGF-1, while BMP-5 increased cell proliferation and cartilage matrix synthesis [119, 120].

 IGF-1, a structural and functional analog of insulin, promotes chondrocyte proliferation and differentiation while it inhibits apoptosis [89, 93]. It is also an important regulator of PTHrP-Ihh feedback loop. The lack of IGF results in downregulation of Ihh expression and upregulation of PTHrP [51]. IGF-1 favors chondrocyte hypertrophic development as it induced type X collagen and alkaline phosphatase in avian sternal chondrocytes [108, 112]. In addition, insulin and IGF-1 [121] both are strong stimulators of aggrecan and type II collagen synthesis [122].

 Furthermore, chondrocyte differentiation in the growth plate is regulated by various transcription factors [123]. Transcription factors Sox9 and -4 have been shown to determine the rate of chondrocyte differentiation into hypertrophy and the expression of chondrocyte-specific matrix molecules including Col2A1, Col9A2, Col11A1, and aggrecan [124–130]. They are also required to prevent conversion of proliferating chondrocytes into hypertrophic chondrocytes [55]. Transcription factors Runx1-3 are the most important as they play a crucial role both in chondrocyte maturation and had been shown to induce MMP-13 expression [77, 80, 125, 131]. Recently, the involvement of several other transcription factors such as Shox/Shox2, Dlx5, and MEF2C has been shown to control skeletal growth that suggests their potential contribution in ectopic chondrocyte hypertrophy development [132, 133]. Wnt/beta-catenin signaling can also mediate chondrocyte hypertrophy as it is capable of upregulating type X collagen, Runx2, and alkaline phosphatase expression while inhibiting Sox9 and type II collagen expression [92].

 Prostaglandin E2 (PGE2), a potent lipid molecule that regulates a broad range of physiologic reactions, can inhibit growth plate chondrocyte differentiation by downregulation of differentiation-related genes COL10A1, VEGF, MMP-13, and alkaline phosphatase expression as well as their enzyme activity [134, 135]. At the same time, low concentrations of this prostaglandin are capable of increasing proliferation of growth plate chondrocytes [136, 137]. In contrast, chemokine stromal sell-derived factor 1, annexin V, and Ank have been shown to stimulate hypertrophy, mineralization, and apoptosis, when they are overexpressed in nonmineralizing growth plate chondrocytes [82, 85, 138, 139]. 

 Extracellular matrix proteins produced by chondrocytes have also exhibited a capacity to regulate growth plate chondrocyte hypertrophy. Thus, type II collagen, aggrecan, and matrilin-3 are likely to inhibit hypertrophy as these matrix component deficiency produced premature maturation in mutant chondrocytes [140–142]. Furthermore a functional link between chondrocyte hypertrophy and extracellular matrix degradation is also supported by the fact that downregulation of chondrocyte hypertrophy evidenced by suppression of type X collagen, Runx2 and MMP-13 expression is associated with inhibition of collagen cleavage activity in cultured hypertrophic growth plate chondrocytes treated with MMP-13 inhibitor [8, 143, 144]. This indicates a functional link between chondrocyte hypertrophy and extracellular matrix degradation.

 It is necessary to note that variable effects of regulatory molecules are carefully coordinated to provide accuracy in the process of endochondral ossification. Thus, it has been demonstrated that growth plate chondrocyte progression to hypertrophy is a subject to negative control that can be arrested at various checkpoints [112]. Accordingly, an early proliferative phenotype in avian fetal chondrocytes has been reassumed by treatment with TGF*β*2, FGF-2, and insulin in combination, while differential Ihh expression was responsible for acquisition of the late proliferative phenotype in hypertrophic cells [112]. In another study, the release of terminally differentiated hypertrophic chondrocytes from their environment also resulted in downregulation of type X collagen synthesis, activation of proliferation, and reinitiation of aggrecan synthesis [145].

 Therefore, chondrocyte differentiation is carefully regulated in the course of endochondral ossification. Eventually, epiphyseal chondrocytes give rise to articular cartilage, whose structural components and regulatory networks at least partially resemble that in the growth plate.

## 4. Zonal Gene Expression in Healthy Articular Cartilage

 Healthy articular cartilage is characterized by a very low expression of collagens type II, VI, IX, and XI [146] and relatively high turnover rate for aggrecan [147]. It is also characterized by expression of matrix turnover genes such as MMP-3 [148], occasionally detected MMP-1, -8, -13 [149], and growth factors TGF*β*1 [150] and PTHrP [24, 151]. Antiangiogenic factor chondromodulin-1 [152, 153], p16INK4*α*, and Gadd45*α*/*β* genes, the latter is associated with environmental and intrinsic stress [154, 155], are expressed in all the cartilage zones. At the same, time no expression of type I and X collagens [156, 157], a complete lack of expression of TGF*β*2, IGF-1, Ihh [158, 159], annexin VIII [160], and osteocalcin [30] was observed in healthy cartilage.

 Articular cartilage can be divided into superficial, mid-, and deep zones; the latter is followed by the calcified cartilage providing junction of the cartilage to the subchondral bone [157]. These zones differ in expression of specific matrix molecules, their modifying enzymes, and regulatory growth factors, which are responsible for articular cartilage integrity and function. Although normal articular chondrocytes are less metabolically active than the growth plate chondrocytes, some similarity in gene expression pattern in the individual cartilage zones has been noted.

 Superficial zone of healthy articular cartilage contains flattened chondrocytes surrounded by specialized extracellular matrix rich in thin collagen fibrils [161] and small leucine-rich proteoglycans-decorin and biglycan [162]. It also contains the lowest amount of predominant cartilage proteoglycan aggrecan compared to other zones of articular cartilage. This zone is rich in regulatory molecules such as TGF*β*1 and -3 and BMP 1–6 [37, 40]. Proliferative potential of these cells is indicated by the expression of cyclin D2; however, it may be suppressed by cell division inhibitors such as growth arrest specific protein (Gas)-1 and Gadd45*α*, which are also expressed in this cartilage zone [37]. This is supported by the lack of superficial chondrocyte proliferative activity determined by PCNA staining [163]. MMP-3 expression was observed in this cartilage zone more often than MMP-1, -8, and -13, however these proteinases do not produce any matrix degradation and are likely involved in matrix turnover [149, 164]. Expression of antiapoptotic Bcl2 and Bag1 genes was detected predominantly in this zone in old mice, while it was observed throughout the articular cartilage in the young animals [63]. 

 Mid-zone chondrocytes are round in shape, surrounded by ECM composed of thick collagen fibrils and rich in aggrecan. Chondrocytes in this zone do not show any proliferative activity determined by PCNA staining similar to superficial zone cells [163]. However, these cells are likely to possess a potential for proliferation, as FGF-2, capable of inducing proliferation in normal articular chondrocytes in culture [165], has been detected in the mid-zone of mouse articular cartilage [49]. BMP1–7 expression was also observed in the mid-zone of normal articular cartilage [40]. 

 Deep zone chondrocytes are grouped in clusters and resemble hypertrophic chondrocytes of the growth plate [3]. In this zone, cartilage matrix has the highest content of aggrecan [166], the lowest amounts of small leucine-rich proteoglycans [162], and the largest diameter of collagen fibrils. Similar to hypertrophic zone of the growth plate, BMP1–7 [40], Ihh expression [167], and the highest amount of annexin VI-positive cells were observed in the deep zone of human articular cartilage [163]. The lowest part of the deep zone, which is partly calcified, expressed a marker of chondrocyte hypertrophy type X collagen and is rich in alkaline phosphatase. MMP-13 expression [149] and negligible activity of chondrocyte apoptosis was also sometimes observed here [168, 169].

 However, in spite of low activity of cellular and matrix turnover, healthy articular cartilage possesses a strong metabolic potential, whose activation is observed during development of pathological condition such as OA.

## 5. Early Development of Osteoarthritis

 Early OA changes in articular cartilage are associated with significant metabolic activation of articular chondrocytes. This involves sequential and zonal upregulation of chondrocyte differentiation-related genes as well as an increase in the activity of the same MMPs, which are responsible for matrix degradation in the hypertrophic zone of the growth plate. Spatially, these genes are upregulated in the mid- and superficial zones of articular cartilage, where lately the first signs of cartilage destruction occur.

 Mild OA changes (Mankin 1–4) are characterized by the loss of proteoglycans in the surface area [163, 170, 171]. Although these changes were not accompanied by significant structural disturbances in the tissue, they were associated with increased type II collagen and aggrecan synthesis, upregulation of chondrocyte proliferation evidenced by increased PCNA and Ki67 staining and MMP-13 expression [172–175]. This was followed by the cellular changes similar to those observed in hypertrophic zone of the growth plate as indicated by type X collagen production, collagenase and alkaline phosphatase staining, and increased type II collagen cleavage activity in the mid-zone [172, 176]. Later, chondrocyte activation extends to the superficial zone, where it is accompanied by chondrocyte apoptosis evidenced by the presence of the cells carrying DNA nicks [172]. IL-1*β* expression is also upregulated both in the superficial and deep cartilage zones in early OA [177]. Similar to biphasic MMP-13 expression in the growth plate, upregulation of this collagenase in the articular cartilage was initially preceded and later accompanied by type X collagen and alkaline phosphatase expression [172, 176].

 An early OA articular cartilage degeneration is observed focally. Spatial distribution of chondrocyte differentiation-related gene expression in the areas adjacent to and remote from the early lesion also resembles that in the growth plate and is associated with increased collagenase cleavage of type II collagen. Thus, collagenases MMP-1, MMP-14 (MT1-MMP), and aggrecanase ADAMTS-5 (but not ADAMTS-4), cytokines IL-1*α*/*β* and TNF-*α*, chondrocyte terminal differentiation-related genes COL10A1, MMP-13, MMP-9, Ihh, and caspase 3 were often upregulated in the vicinity of the lesion. Growth factors associated with growth plate chondrocyte proliferation, namely, FGF-2, PTHrP, and TGF *β*1/2, as well as the matrix molecules COL2A1 and aggrecan, were expressed adjacent to and remote from the lesion [22]. In addition, a distinct spatial reorganization in human superficial chondrocytes in remote area from early OA lesions has been recently reported [178]. 

 However, of all genes, only caspase 3 and ADAMTS-5 expression was exclusively seen in association with early lesions. Elevation of collagenase activity was associated with a frequent elevation of expression of COL10A1, caspase 3, IL-1*α*/*β*, MMP-1, and ADAMTS-5 and a decreased expression of Sox-9, TGF-*β*1, TGF-*β*2, TNF-*α*, and aggrecan [22]. 

 Moderate OA changes in the articular cartilage (Mankin 6–9), which are characterized by the lack of fibrillations, some loss of superficial zone, and some clustering of cells [163, 171], are associated with the increase of PCNA staining in the superficial zone and annexin VI and VIII antigen upregulation in the mid- and deep zones [160, 163, 179]. 

 Therefore, articular chondrocyte activation in early OA, which is the most pronounced in the superficial and mid-zones, resembles that observed during chondrocyte maturation in the growth plate.

## 6. Gene Expression in Late Osteoarthritic Cartilage

 Severe OA (Mankin ≥10) is characterized by extensive fissuring and fibrillation, clustering of chondrocytes, and loss of cartilage [163]. Cartilage zonal organization is disturbed. The superficial zone degradation produces rough fibrillated surface, fissures, and cracks extending to the calcified zone. This is accompanied by severe proteoglycan loss followed by degradation of type II collagen [164, 180]. Collagen degradation occurs around chondrocytes. At this time, upregulation of MMPs-13, -2, -11, ADAMTS as well as expression of collagens type I, II, III, VI, and X were observed near the articular surface [148, 181–183] and was accompanied by strong expression of IL-1*β* and TNF*α* [149]. At the same time, collagen replenishment is limited as Col2A N-propeptide, a marker of collagen synthesis, was detected only in the deep zone close to subchondral bone [184]. As it was stated above, all these gene activities have been also observed in the hypertrophic zone of the fetal growth plate. 

 Chondrocyte terminal differentiation-related gene expression is also observed in cell clusters located around fissures [185, 186]. In these clusters, both collagen type II and X synthesis [187] as well as TGF*β*3 and its receptor regulator Smad-2P expression were observed [188]. 

 PCNA and syndecan-3, the markers of early fetal chondrocyte differentiation, as well as annexin VI and alkaline phosphatase, which are involved in terminal stage of differentiation, all are upregulated near articular surface in human OA articular cartilage [30, 163]. At the same time, annexin VIII and osteocalcin, which were never detected in normal articular cartilage, were observed in the mid- and deep zones in late OA cartilage [30, 160]. An increase in BMP-2 expression [188] was associated with upregulation of tumor suppressor p53 expression [189] and cyclin-dependent kinase inhibitor p16INK4a upregulation in all the cartilage zones [155] indicating inhibition of proliferative potential in late OA chondrocytes. However, repression of antiproliferative factor Tob1 has been also reported in the late stage of knee OA cartilage [183].

 The most severely damaged rodent knee OA articular cartilage has shown significantly reduced expression of proliferation-related growth factors and their signaling molecules such as PTHrP, TGF*β*3 and Smad-2P, TGF*β*1 and its receptor II [188, 191]. However, in human hip OA, both downregulation and upregulation of TGF*β*1–3 isoform expression compared to healthy cartilage have been reported [43, 192], while one study failed to detect any upregulation of chondrocyte differentiation and hypertrophy markers associated with late OA [193].

 Antiangiogenic factor chondromodulin-1 downregulation concomitant to VEGF upregulation indicating increased vascular invasion into cartilage in advanced OA has been also observed [194, 195]. This was accompanied by upregulation of chondrocyte apoptosis, a marker of the final step of chondrocyte differentiation, which was more pronounced in OA cartilage compared to normal specimens [189].

 Overall degrading activity prevailed over synthesis as serum levels of Col2A N-propeptide were lower than that of collagen degradation products in late OA patients compared to controls indicating the uncoupling of collagen synthesis and degradation in OA [190]. Moreover, serum increase in both Col2A N-propeptide and collagen degradation fragments was often indicative on the most aggressive disease progression [196].

 Thus, the similarity in the gene expression profiles associated with matrix destruction in OA articular cartilage and in the hypertrophic zone of the growth plate observed in the majority of studies suggests an acquisition of hypertrophic phenotype traits by OA articular chondrocytes.

## 7. Inhibition of Articular Chondrocyte Hypertrophy Suppresses OA Cartilage Degeneration

 The similarity of ECM degradation in OA to that in the hypertrophic zone of primary growth plate involves upregulation of type II collagen cleavage by collagenase and expression of regulatory differentiation-related growth factors and matrix proteins, which are associated with chondrocyte hypertrophy [6, 22, 25, 28]. Therefore, the above observation that hypertrophic changes in the growth plate chondrocytes are reversible [112] suggests a possibility for OA articular chondrocytes to regain healthier phenotype when they are treated by the agents inhibiting fetal hypertrophy.

 In fact, the same growth factors, namely, TGF*β*2, FGF-2, and insulin, which were previously used individually or in combination to suppress hypertrophy in growth plate chondrocytes [112], have been shown to be capable of arresting type II collagen cleavage, chondrocyte differentiation-related gene, and proinflammatory cytokine expression in human OA articular cartilage explants [197]. Another combination of TGF*β*1 and IGF1 inhibited collagen degradation, a marker of extracellular matrix destruction, which was induced by oncostatin M and TNF*α* in bovine articular cartilage [198]. It has been also shown that TGF*β* inhibition of chondrocyte differentiation is likely mediated by Smad2/3 pathway through modulation of Runx2 function [75]. In another study, a major proliferation-related growth factor PTHrP downregulated terminal differentiation-related genes in cultured mineralizing articular chondrocytes from the deep zone as well as in chondrogenic articular cartilage constructs [199, 200]. It is worth to note here that growth factors, which were capable of inhibiting collagen degradation in OA articular cartilage, are predominantly expressed in the proliferative zone of the growth plate and are required for chondrocyte proliferation in the development. 

 Similar effect on suppression of collagen cleavage in association with inhibition of chondrocyte hypertrophy-related genes and proinflammatory cytokines IL-1*β* and TNF*α* has been observed on treatment of OA explants with low concentrations of PGE2 [201]. Although PGE2 is expressed in all the growth plate zones, it is primarily required for fetal chondrocyte proliferation [137] and is capable also of inhibiting their terminal differentiation [134, 135] and expression of proinflammatory mediators [202]. At the same time, PGE2 at higher concentrations has been shown to exert stimulating effects on cartilage degradation [203]. 

 Alternatively, downregulation of the genes, which are expressed in the hypertrophic zone of the growth plate and associated with chondrocyte terminal differentiation such as Hedgehog signaling, TGF*β*1/BMP signaling, transforming growth factor-beta-activated kinase (TAK) 1, cyclin-dependent kinase inhibitor p16INK4a, ADAMTS5, RUNX2, and caspases, resulted in abrogation of matrix degeneration, less type X collagen production, and MMP-13 expression [155, 204–210]. Therefore, upregulation of the genes associated with growth plate chondrocyte proliferation or downregulation of hypertrophy-related genes favors acquisition of healthier phenotype in OA articular chondrocytes. 

 However, while direct inhibition of cartilage degradation by the agents capable of regulating chondrocyte differentiation is an attractive means to counteract articular cartilage degeneration in OA, it has several limitations. Thus, following the inhibition of cartilage degradation by individual growth factors (GFs) reparation of articular cartilage in OA in vivo may require a combination of GF [18, 211, 212]. For example, being the most efficient in suppressing OA articular cartilage destruction [197], TGF*β*2 alone may not be capable of restoring the anabolic functions of healthy articular cartilage since it has been reported to downregulate type II collagen and aggrecan synthesis [108, 213]. In contrast, in responsive individuals, insulin may facilitate tissue repair as it is a principal anabolic agent in the articular cartilage [214]. FGF-2 can also promote cartilage repair [212] by itself or inducing local TGF*β* or its own expression [215]. In addition, combinations of these and other growth factors have been shown to produce synergistic effect in maintaining synthesis of matrix molecules in articular and growth plate chondrocytes [108, 216, 217]. 

 Another concern on GF application is related to their possible catabolic effects. Although no evidence has been obtained that TGF*β*2 can act catabolically in human OA articular cartilage [197], destructive potential of this growth factor at high concentration was observed in normal articular cartilage in vivo after its intraarticular injections, which produced joint swelling, fibroblastic proliferation of synovial membrane, and profound loss of articular cartilage proteoglycan in rabbit joints [213]. Therefore, the delivery of exact therapeutic amount of the growth factor to the site of articular cartilage destruction may be important. This has been demonstrated in a recent study, where deleterious effect of TGF*β*1 capable of inducing synovial fibrosis has been counteracted by combined overexpression of TGF*β*1 and its inhibitor Smad7 [188]. This resulted both in prevention of proteoglycan (PG) loss and in increase in PG content in mouse OA cartilage.

## 8. Modeling of OA-Related Changes in Healthy Articular Cartilage Is Associated with Chondrocyte Hypertrophy Development

 In healthy adult articular cartilage, chondrocyte differentiation does not occur in the noncalcified cartilage [218]. However, when maturational arrest is abolished, chondrocyte differentiation-related genes, which are barely expressed in healthy articular cartilage, become upregulated followed by hypertrophic changes in the cells and extracellular matrix. If this notion is true, stimulation of degradation in healthy articular cartilage should be accompanied by chondrocyte hypertrophy development. The relieve of transcriptional repression can be attained by cartilage treatment with azacytidine C (Aza-C), which replaces cytidine bases in genomic DNA during replication and disturbs methylation pattern of cytidines (CpG islands) in target gene promoters. This was associated with upregulation of PTHrP, governing chondrocyte proliferation in the growth plate, as well as chondrocyte hypertrophy-related collagen type X, Ihh, and alkaline phosphatase gene expression, and the increase in chondrocyte cell size in healthy articular cartilage [219–221].

 On the other hand, alterations associated with chondrocyte hypertrophy in the growth plate are always accompanied by overt extracellular matrix resorption producing its degradation fragments [3]. Therefore, it is not surprising that collagen and/or fibronectin degradation peptides, which can be also released on mechanical destruction of articular cartilage caused by trauma or joint overload in case of anterior cruciate ligament transection, have been shown to be capable of inducing articular cartilage degradation by upregulation of collagenase and MMPs activity [222–224]. Besides, these peptides upregulated chondrocyte proliferation, production of type X collagen and apoptotic cells on the surface of articular cartilage explants [172, 224]. Collagen fragments may also account for OA-like changes induced in healthy cartilage by overexpression of MMP-13, which were associated with chondrocyte hypertrophy in mouse articular cartilage [47]. In addition, other matrix disturbances such as lack of matrilin-3 by a corresponding gene knockout produced premature chondrocyte maturation to hypertrophy and formed predisposition to develop severe OA in mice [141].

 Functional disturbances in the regulatory genes involved in chondrocyte differentiation can also produce OA-related changes in healthy articular cartilage resembling chondrocyte maturation in the growth plate. Thus, deficiency in TGF*β* signaling, which is essential for articular cartilage maintenance and had been induced either by overexpression of functionless TGF*β* type II receptor [225] or by deletion of Smad3 signaling [104, 226], caused accelerated chondrocyte differentiation associated with type X collagen expression and OA-like changes in articular cartilage. It has been also shown that the absence of signaling through Fgfr (fibroblast growth factor receptor) 3 in the joints of Fgfr3(−/−) mice produced premature cartilage degeneration and early arthritis [227]. In contrast, TGFalpha signaling suggests catabolic potential of this growth factor as it has been shown to stimulate articular chondrocyte proliferation, formation of cell clusters followed by expression of matrix-degrading enzymes MMP-13, cathepsin C and downregulation of Sox9, as well as collagen and aggrecan expression in rat articular osteochondral explants [228].

 Being an important factor of OA articular cartilage pathology proinflammatory cytokines such as TNF*α* and IL-1*β* have been shown to mediate articular cartilage degradation by upregulation of matrix-degrading MMPs [229]. It has been observed recently that increased expression of proinflammatory agents such as TNF*α*, chemokines IL-8, growth-related oncogene *α* (GRO*α*), or the multiligand receptor for advanced glycation end products (RAGE) induced also chondrocyte hypertrophy evidenced by collagen type X expression [230, 231]. This suggests a link between inflammation and altered differentiation in articular chondrocytes [231]. Interestingly, the impairment TGF*β* signaling by IL-1*β* was mediated by downregulation of TGF*β*RII [232]. The loss of function of this receptor has previously been linked to chondrocyte hypertrophy induction and OA development in animal studies [225]. 

 Therefore, OA-like alterations in healthy articular cartilage induced by the mediators, which are upregulated in the hypertrophic zone during endochondral ossification, are accompanied by ECM degradation and associated with articular chondrocyte hypertrophy.

## 9. Conclusions

 The data presented here shows a significant progress in our understanding of molecular mechanisms of articular cartilage degradation in OA. They involve at least in part similar machinery of extracellular matrix resorption in the hypertrophic zone of the growth plate and in OA articular cartilage in the course of its degeneration. The observation that profound cellular phenotypic changes in articular chondrocytes occur prior the overt cartilage matrix degradation monitored histologically suggests that articular chondrocyte phenotype modifications can be recognized very early in the disease at gene expression level favoring timely disease recognition, which could help its prevention. This implies also innovative opportunities in suppression of cartilage matrix degradation targeting inhibition of chondrocyte hypertrophy and suggests new targets for therapeutic intervention. For this purpose, further studies are required in search of new agents generating programmable articular chondrocyte phenotype modification.

## Abbreviations

OA: Osteoarthritis
ECM: Extracellular matrix
MMP: Metalloproteinases
IL: Interleukin
ADAMTS: A disintegrin and metalloprotease with thrombospondin motifs
PTHrP: Parathyroid hormone-related peptide
TNF: Tumor necrosis factor
ALP: Alkaline phosphatase
PCNA: Proliferating cell nuclear antigen
SLRPs: Small leucine-rich proteoglycans
BMP: Bone morphogenetic protein
FGF: Fibroblast growth factor
TGF: Transforming growth factor
TGFR: Transforming growth factor receptor
IGF: Insulin growth factor
Bcl-2: B-cell lymphoma 2
Sox9: SRY-type high-mobility-group box transcription factor-9
GADD45beta: Growth arrest and DNA damage-inducible 45beta
Bag1: Bcl2-associated athanogene 1, a Bcl2-binding protein capable of enhancing Bcl2 activity
COL10A1: Type X collagen
BMP: Bone morphogenetic proteins
CTGF: Connective tissue growth factor
VEGF: Vascular endothelial growth factor
Ihh: Indian hedgehog
RUNX2: Runt-related transcription factor
COL2A1: Collagen type II
Ank: Ankylosis protein
PGE2: Prostaglandin E2
Gas1: Growth arrest specific protein1
Aza-C: Azacytidine C
Fgfr3: Fibroblast growth factor receptor 3
RAGE: Multiligand receptor for advanced glycation end products
GRO: Growth-related oncogene
PG: Proteoglycan
TAK: Transforming growth factor-beta-activated kinase
GF: Growth factor.
